# Efficiency, Safety, and Efficacy of High-Power Short-Duration Radiofrequency Ablation in Patients with Atrial Fibrillation

**DOI:** 10.1155/2021/8821467

**Published:** 2021-02-15

**Authors:** Xuerong Sun, Jiang Lu, Jinxuan Lin, Tianjie Feng, Ni Suo, Lihui Zheng, Zhimin Liu, Gang Chen, Xiaohan Fan, Shu Zhang, Guodong Niu

**Affiliations:** ^1^Arrhythmia Center, State Key Laboratory of Cardiovascular Disease, Fuwai Hospital, National Center for Cardiovascular Diseases, Chinese Academy of Medical Sciences, Peking Union Medical College, Beijing 100037, China; ^2^Arrhythmia Center, Fuwai Yunnan Cardiovascular Hospital, Kunming 650102, China

## Abstract

Pulmonary vein isolation (PVI) is the cornerstone therapy of atrial fibrillation (AF). Radiofrequency catheter ablation (RFCA) is performed using a point-by-point method to achieve durable PVI. However, this procedure remains complex and time-consuming, and the long-term clinical outcomes are still not satisfactory. Recently, there has been increasing interest in the clinical application of high-power short-duration (HPSD) approaches in the field of RFCA. HPSD ablation, distinguishing it from the conventional ablation strategy, delivers RF energy at a high power and saves the dwell time at each site. It is unknown whether the HPSD approach can bring some gratifying changes in the field of RF energy ablation. A number of experimental studies and clinical studies have been conducted regarding this topic. The review aimed to summarize the research findings and evaluate the procedural efficiency, safety, and clinical outcomes of the HPSD approach based on the evidence available to date.

## 1. Introduction

Pulmonary vein isolation (PVI) is the cornerstone of current ablation strategies for the treatment of symptomatic atrial fibrillation (AF) [1–3]. Pulmonary vein (PV) reconnection is the major reason for AF recurrence [4, 5]. Durable PVI can be performed by radiofrequency catheter ablation (RFCA) using a point-by-point method [5]. However, this process is complex and time-consuming, especially when compared with cryoballoon ablation (CBA) and pulse field ablation (PFA) [3, 6]. Additionally, procedure-related complications, such as esophageal damage, stream pops, and cardiac tamponade, still remain a concern [3]. Thus, technological renovation is required to improve procedural efficiency, efficacy, and safety in the field of RFCA.

Recent published data showed that RF energy can be delivered at 45 to 90 W for shorter durations aiming for contiguous lines and transmural lesions, especially owing to the development of novel open-irrigated catheters [7–9]. High-power short-duration (HPSD) approach is different from the conventional ablation therapy (CAT) at the ablation settings. CAT is usually performed at 25 to 40 W delivered for 20 to 60 s [10, 11]. Theoretically, the HPSD approach appears feasible and leads to an efficient PVI by shortening the RF application. However, it remains a considerable concern whether the HPSD approach can achieve comparable or superior outcomes without compromising safety. A number of experimental and clinical studies have been conducted to evaluate its efficacy and safety. This article reviews the experimental findings and clinical outcomes of HPSD ablation.

## 2. In Vivo and In Vitro Ablation Studies on HPSD

An in vitro study on porcine left ventricular myocardium indicated that RF delivery at a higher power (40 to 50 W), shorter duration (5 to 10 s), and low irrigation flow rate (2 ml/min) with 20 g contact force (CF) was anticipated reliably to create transmural lesions of the posterior left atrial wall in patients with AF [12]. Steam pops were observed usually when the irrigation flow rate was 2 mL/min, power 40–50 W, and ablation duration 20–40 s. Meanwhile, the tissue temperature monitoring at a 5 mm depth with HPSD (50 W for 5 s) was lower than 50°C (the temperature at which irreversible injury happens), which suggested that HPSD RF ablation may be safer in preventing collateral damage to neighboring structures. Another study based on in vitro and in vivo sheep models observed that an ablation setting of 50–80 W for 5 s adequately achieved the similar lesion depth of 2-3 mm (atrial wall thickness based on a human autopsy series) with 10 g CF at an irrigation flow rate of 30 ml/min [13]. In addition, an ablation setting of 50–60 W for 5 s reduced the incidence of steam pops and collateral damage to neighboring structures compared with the conventional ablation setting of 40 W for 30 s.

**Figure 1:**
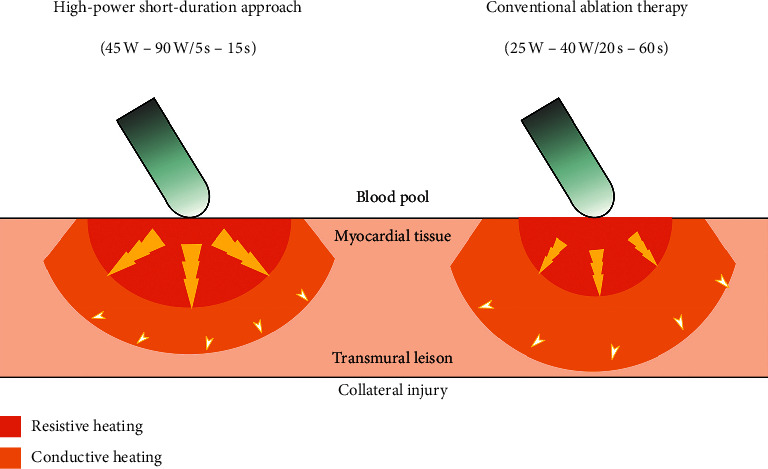
Comparison between HPSD approach and CAT. Lesion dimensions of radiofrequency ablation and heating distribution in myocardial tissue are demonstrated. HPSD approach and CAT could create comparable lesion size. HPSD approach results in an ablation lesion that is heated directly from the catheter during the resistive phase (red part), while, in CAT, myocardial tissue is largely heated because of conductive heating (golden yellow part).

Recently, a novel open-irrigated catheter, QDOTMICRO^TM^ catheter, developed on a similar platform of the Thermocool SmartTouch SF catheter, with 56 irrigation holes through the 3.5 mm tip for an improved irrigation system and six symmetrical temperature sensors for accurate real-time temperature monitoring at the catheter-tissue interface, contributed to the clinical application of a very high-power ablation setting of 90 W for 4 s [10]. Previous in vivo studies that were performed showed that, compared with CAT, for a single ablation lesion and linear atrial lines, an ablation setting of 90 W for 4 s resulted in larger lesion diameters, similar depth, and contiguous lines without apparent gaps, ensuring better contiguity and transmurality [11]. It was shown that this HPSD approach modified the relationship between resistive and conductive heating, avoiding potential collateral damage to adjacent structures by limiting conductive heating (Figure 1). The results of the in vitro and in vivo studies on HPSD ablation are summarized in Table 1.

## 3. HPSD Ablation Setting and Lesion Endpoint

The ideal HPSD ablation setting should be determined according to the requirements of the lesion transmurality, linear ablation contiguity, and safety issues, including but not limited to RF power, duration, CF, irrigation flow rate, impedance drop, catheter stability, temperature measurement, and interlesion distance (ILD) [5]. Because of the limitation that HPSD ablation lesions vary greatly in a range of rather short RF durations, identifying a valid parameter capable of predicting the lesion size is essential. There have been various lesion endpoints of energy delivery at each site developed in different studies, including the combination of RF power and dwell time, unipolar signal modification, impedance drop, loss of pacing during RF delivery, and some defined ablation lesion markers such as lesion size index (LSI) and ablation index (AI) [14–19].

HPSD RF ablation was usually delivered at a high or very high power of 50–90 W for less than 10 s. The combination of RF power and dwell time (90 W for 4 s, 50 W for 5–7 s, etc.) was used as a common lesion endpoint to predict the lesion volume. A quantitative ablation strategy was developed into a proper ablation lesion marker composed of important lesion information. Force‐time integral (FTI), an integral of CF and RF duration, was previously used for AF ablation but did not contain RF power, which had a considerable contribution to the ablation lesion volume [14]. Other than FTI, LSI and AI, including CF, RF duration, and RF power in different weighted formulas, were estimated as convincing ablation lesion markers for guiding RF ablation and decreasing the long-term PV reconnection rate [15, 20]. It was observed that the CLOZE standard of AI targets of ≥400 for the posterior wall and ≥550 for the anterior wall with 6 mm ILD was used to guide PVI in paroxysmal AF ablation and can achieve excellent results of 92.3% single-procedure 12-month freedom [5]. Some studies also showed that high power (40–50 W) ablation guided by AI appeared to be a feasible, effective, and safe technique for PVI in patients with AF. However, AI is still not a reliable marker for the HPSD approach. It was commonly observed that, at high power delivered, the initial AI value displayed is already >400, probably due to time-consuming confirmation of catheter stability. In addition, as shown by Chinitz et al. [16], an impedance drop of more than 10 Ω during ablation was associated with a durable transmural lesion. It was also suggested that the complete elimination of the negative component of the unipolar atrial signal (a change from the initial RS pattern to the final R pattern) reflects an established transmural lesion [17–19]. The HPSD RF ablation settings and lesion endpoints in published clinical studies are demonstrated in Table 2 [21–35].

In addition, to reduce conductive heat transfer and avoid collateral injury to surrounding structures, it is recommended to reduce the application of total energy at the posterior left atrial wall (shorter duration or lower RF power compared with the anterior wall) when using the HPSD approach.

## 4. Procedural Efficiency

The RF ablation time with HPSD approach is remarkably reduced compared with conventional ablation, which is confirmed by all the published studies on HPSD ablation versus CAT [24–27, 29–32]. Only two out of nine studies [21, 24, 26, 27, 29–32, 34] showed that the procedure time was not remarkably shorter in the HPSD group than in the CAT group. The procedural efficiency is mainly due to the reduction of RF dwell time at each site with high-power delivery. The reduction in mean procedure time was less than the sole reduction in mean RF ablation time. It was reported that the mean procedure time during HPSD ablation was reduced by 12–39%, while the mean RF ablation time was reduced by 36–65%. RF ablation time and procedure time were saved mainly owing to the high-power delivery and the improved first-pass isolation rate. As for the fluoroscopy time, 50% of studies (four out of eight) [21, 24–27, 30–32, 34] showed that HPSD ablation took a shorter fluoroscopy time than CAT and that heterogeneity might be associated with the operator' s experience, HPSD approach workflow, different mapping and ablation techniques, a wide range of ablation power, and different ablation strategies.

The procedural efficiency of HPSD ablation has a number of benefits. Shorter procedures limit patient exposure to anesthesia and intravenous fluids with lower procedure-related complication rates, especially for patients with potential heart failure, possibly contributing to a safer procedure. Decreased fluoroscopy time is beneficial to patients, operators, and supporting staff. Most importantly, when a shorter RF dwell time at each site is applied, catheter stability is much easier to maintain, which makes it much more suitable for unskilled operators. Clinical efficiency between HPSD group and CAT group was compared in Table 3.

## 5. Clinical Efficacy

For the acute PVI outcomes, four out of five studies [25, 26, 29, 30, 33] reported that the HPSD approach contributed to a significantly higher first-pass ipsilateral PVI rate, while three out of five studies [25, 29–31, 34] observed a significantly lower acute PV reconnection rate compared with CAT, which can also help reduce the RF ablation time to achieve complete PVI and improve procedural efficiency. Table 3 summarized the clinical efficacy between HPSD group and CAT group.

With regard to long-term outcomes, it is controversial whether HPSD ablation can achieve superior clinical outcomes compared with conventional ablation. Five studies [21, 24, 27, 31, 34] reported the one-year AF recurrence rate between HPSD ablation and conventional therapy. It was shown that, in the study population with only PAF, the AF recurrence rate was remarkably lower in the HPSD group than in the CAT group, while, in the study population with combined PAF and PsAF, the HPSD group did not have dominant advantages over the CAT group. The variety of AF types and ablation strategies used may have contributed to this. Until now, the longest follow-up duration between HPSD ablation and conventional ablation is three years, showing that the recurrence AF rate was similar (26.5% vs. 30.7%; *P*=0.23) [21].

Shin et al. [34] performed a randomized controlled trial and found that the 12-month freedom rate from AF was not significantly different between the HPSD (50 W) and conventional groups (*P*=0.862). They also reported on the risk factors for AF recurrence. In the multivariate analysis, non-PAF (hazard ratio [HR] 2.836, *P*=0.045) and AI (HR 0.983, *P*=0.001) were independent risk factors for AF recurrence.

## 6. Safety and Complications

There is a major concern for procedure-related complications with the HPSD approach and whether the procedural efficiency and efficacy outcomes come at a cost of safety. Experimental studies demonstrated that the HPSD approach created a shallower and broader lesion but appeared to have a narrow safety and efficacy window [10–13]. Thus, the RF duration for the HPSD approach is the critical determinant of lesion formation. When the ablation is insufficient, superficial and nontransmural lesions are created; when the ablation is excessive, collateral damage (including injury to the esophaus annd phrenic nerve), steam pops, and subsequent pericardial tamponade could ooccur. Silent and clinical stroke, as a result of thrombus formation, may also occur.

Wrinkle et al. [36] reported extremely low complication rates and a very low incidence (0.0087%) of atrioesophageal fistulas with HPSD approach for 45–50 W in a cohort of 11,436 patients in four experienced centers. Bunch et al. [21] observed one symptomatic esophageal ulcer in 402 patients undergoing HPSD ablation with 50 W for 2–5 s in their early experience. Chen et al. [23, 37] and Kaneshiro et al. [38] used AI (400) guiding HPSD (45–50 W) for the left atrial posterior wall and combined multisensory luminal esophageal temperature monitoring and postablation esophageal endoscopy. Chen et al. [23, 37] observed that the incidence of luminal esophageal temperature >39°C was 47% (57/122), and the rate of endoscopically detectable lesion formation was 2 of 57 (3.5%) without evident clinical sequelae. Kaneshiro et al. [38] compared HPSD ablation with CAT and found that the incidence of esophageal thermal injury was significantly higher in the HPSD group (37% versus 22%; *P*=0.011), but the prevalence of esophageal lesions did not differ between the two groups (7% versus 8%). In a study by Reddy et al. [28], esophageal ulcer hemorrhage was observed in one of 52 patients with RF application of 90 W for 4 s via postprocedural endoscopy on day 1, but it resolved with medication. In addition, esophageal injury observed using the postablation late gadolinium enhancement magnetic resonance imaging (MRI) in the HPSD approach (50 W for 5 s) was reported to be similar in incidence and size to that in the CAT group, and there was no evidence of atrioesophageal fistula in either group [39]. Esophageal temperature spatial and temporal characteristics during HPSD ablation with 50 W for 6 s were evaluated using a 12-point esophageal temperature probe. It was reported that all lesions with significant luminal esophageal temperature increase (>2°C) within 20 mm distance from a temperature sensor to a preexisting RF lesion returned to baseline temperature (±1°C) within 60 s after the cession of RF application, indicating that esophageal injury may be avoided if RF was not applied within 20 mm apart from a prior lesion for at least 60 s [40].

With regard to systemic emboli formation and steam pops, published studies cannot provide meaningful data on the risks because of the small volume of study patients. Reddy et al. [28] reported that, in six patients (11.5%), silent cerebral lesions were detected but were not associated with neurologic deficits. Although most disappeared on repeat MRI after one to three months, whether potential impacts remained was uncertain. As for steam pops, Chen et al. [23, 37] reported its occurrence in four patients (3%; 4/122) who underwent anterior wall ablation with 50 W for 550AI in the very first 50 cases, either because of high CF or long ablation duration, and no pericardial effusion or cardiac tamponade was observed. Castrejón-Castrejón et al. [30] observed steam pops in four patients (8.3%) under HPSD ablation with 50–60 W, and none were observed in the conventional group. In Yavin et al.'s study [33], the incidence of steam pops in HPSD (45–50 W, 8–15 s) group was similar to the conventional group (0.07% vs. 0.03%; *P*=0.18). In both studies, no related clinical injury occurred. It seems that the incidence of steam pops is increasing to some extent, but they have not been found to result in clinical injuries.

## 7. Limitations and Future Perspectives

A number of experimental and clinical studies have revealed the efficiency, safety, and efficacy results of the HPSD approach in patients undergoing AF ablation. HPSD ablation is reported to have equivalent or superior procedural efficiency and clinical outcomes compared with conventional ablation. With regard to safety issues, published data cannot provide powerful and substantial evidence, mainly limited to the sample size. It is still worthwhile to anticipate whether the HPSD approach is a game-changing technique for patients with AF.

There has been no comparison of HPSD ablation with CBA or PFA. CBA is performed in a single-step mode, leading to necrosis by freezing and thawing [3]. It is noninferior to RFCA with respect to efficacy and overall safety. With regard to procedural efficiency, CBA requires shorter procedure time (124.4 ± 39.0 vs. 140.9 ± 54.9 min) but longer fluoroscopy time (21.7 ± 13.9 vs. 16.6 ± 17.8 min) than the conventional RFCA strategy [3]. Comparison between CBA and HPSD RF ablation is still lacking. PFA uses high-voltage-pulsed electrical fields to ablate myocardium through the mechanism of irreversible electroporation [6]. A single PFA delivery is accomplished within a heartbeat and it is estimated that the time required to deliver the PFA energy for complete PVI amounted to no more than 3 min per patient [6]. In contrast with HPSD RF ablation, the effects of PFA are almost instantaneous.

Until now, only one randomized controlled trial has been performed, but it does not adequately prove the purported benefits of the HPSD approach, especially in clinical efficacy. A standard HPSD approach is not defined, including RF application settings, local endpoint, workflow, and so on. HPSD ablation is currently used by experienced operators and centers [41, 42]. In addition, long-term outcomes are required to verify its clinical benefits. Some studies have worked on the HPSD approach by compromising high power for a high-irrigation profile, which requires more evidence to clarify its safety. All the aforementioned critical questions need to be answered adequately before the widespread adoption of the HPSD approach.

## 8. Conclusions

RF energy ablation delivered at a high power (45–90 W) for a short duration remarkably improves the procedural efficiency. Compared with CAT, HPSD ablation probably contributes to a higher rate of first-pass isolation and a lower acute PV reconnection rate. Current studies have not provided substantial evidence on long-term outcomes and safety profiles. Further research on the HPSD approach is required to estimate its safety and efficacy, compared with CAT.

## Figures and Tables

**Table 1:** In vitro and in vivo studies on HPSD ablation.

Study	In vitro or in vivo model	Catheter type	Ablation setting (W/s)	Irrigation flow rate (ml/min)	Contact force (g)	Lesion depth (mm)	Lesion width (mm)	Stream pops (%)
Bhaskaran et al. [12]	In vitro and in vivo model: sheep RA myocardium	Thermocool catheter (in vitro)	In vitro model:	30	10	In vitro model:	In vitro model:	—
			40/30			2.7	5.2	
			40/5			1.3	4.1	
			50/5			2.2	5.1	
			60/5			2.4	5.4	
			70/5			2.6	5.9	
			80/5			2.9	6.5	
		Thermocool SmartTouch catheter (in vivo)	In vivo:	30	10	In vivo:	In vivo:	In vivo:
			40/30			2.4	9.1	2/19 (10.5)
			50/5			2.3	7.6	0/18 (0)
			60/5			2.2	7.7	0/14 (0)
			70/5			2.1	7.5	1/12 (8)
			80/5			2.4	8.3	1/9 (11)

Ali-Ahmed et al. [13]	In vitro model: porcine LV myocardium	Thermocool SmartTouch catheter	20/5	2	20	1.4	5.1	—
			30/5			2.1	5.7	
			40/5			2.4	6.6	
			50/5			2.9	7.2	
			20/5	17		1.7	4.2	
			30/5			1.7	5.9	
			40/5			2.6	6.4	
			50/5			3.0	7.3	
			20/10	2		2.6	6.7	
			30/10			3.0	7.9	
			40/10			4.1	8.7	
			50/10			5.0	9.1	
			20/10	17		1.9	5.6	
			30/10			2.8	6.9	
			40/10			3.7	7.8	
			50/10			4.9	7.9	

Barkagan et al. [10]	In vitro model	Thermocool SmartTouch SF catheter (conventional ablation) QDOTMICRO^TM^ catheter (HPSD ablation)	30/30	8	10–20	Transmural	Posterior line	0
							7.9	
							9.5	
							Anterior line	
							3.6	
							8.0	

Leshem et al. [11]	In vitro model: swine thigh muscle	Thermocool SmartTouch SF catheter (conventional ablation) QDOTMICRO^TM^ catheter (HPSD ablation)	25/20	8	10	3.74	6.3	0/28 (0)
			90/4			3.62	10.36	0/28 (0)
			90/6			4.01	10.57	2/28 (7.2)
			70/8			4.32	10.79	1/28 (3.6)
	In vivo model: swine RA/RV myocardium	QDOTMICRO^TM^ catheter	90/4	8	15	RA 3.39	RA 7.0	3/174 (1.7)
						RV 3.82	RV 6.92	

HPSD: high-power short-duration; LV: left ventricle; RA: right atrium; RV: right ventricle.

**Table 2:** The HPSD RF ablation settings and lesion endpoint in published clinical studies.

Study	Study type	Sample size	AF type	Catheter type	High power and dwell time		Contact force	Irrigation flow rate	Lesion endpoint
					Anterior wall	Posterior wall			
Bunch et al. [21]	Controlled study	804	PAF and PsAF	Force-sensing catheter and non-force-sensing catheter	50 W/5–15s	50 W/2–5 s	5-2 g	30 ml/min	Local electrogram and dwell time
Winkle et al. [22]	Cohort study	1,250	PAF and PsAF and long-standing AF	Force-sensing catheter	50 W	50 W	10–40 g	—	Impedance drop, loss of pacing capture, and LSI
Chen et al. [23]	Cohort study	50	PAF and PsAF	STSF catheter	50 W	50 W	Minimum force 3 g	20 ml/min	AI
Vassallo et al. [24]	Controlled study	76	PAF and PsAF	Force-sensing catheter	50 W/6-8 s	45 W/6-8 s	10–20g(anterior)/5–10g(posterior)	35 ml/min	Impedance drop
Okamatsu et al. [25]	Controlled study	60	PAF and PsAF	STSF catheter	50 W	40 W	Minimum force 3g	—	AI
Berte et al. [26]	Controlled study	174	PAF and PsAF	STSF catheter	45 W	35 W	—	15 ml/min	AI
Kottmaier et al. [27]	Controlled study	197	PAF	Flexibility SE catheter	70 W/7 s	70 W/5 s	—	20 ml/min	Power and dwell time
Reddy et al. [28]	Cohort study	52	PAF	STSF catheter	90 W/4 s	90 W/4s	5–30 g	8 ml/min	Power and dwell time
Pambrun et al. [29]	Controlled study	100	PAF	Force-sensing catheter	50 W	40 W	Minimum force 10 g	—	Unipolar signal modification
Castrejón-Castrejón et al. [30]	Controlled study	95	PAF and PsAF	Force-sensing catheter	50–60 W	50–60 W	10 g	—	AI/LSI
Ejima et al. [31]	Controlled study	120	PAF	STSF catheter	50 W	50 W	10g	—	Unipolar signal modification
Yazaki et al. [32]	Controlled study	64	PAF	STSF catheter	50 W	50 W	—	—	Unipolar signal modification and dwell time
Yavin et al. [33]	Controlled study	224	PAF and PsAF	STSF catheter	45–50/15 s	45–50/8s	—	17 ml/min	Impedance drop
Ücer et al. [35]	Cohort study	25	PAF and PsAF	STSF catheter	50	50/6–10 s	10–15 g (posterior)/15–20 g (anterior)	15	Power and dwell time
Shin et al. [34]	RCT	150	PAF and PsAF	STSF catheter	50	50	20g	15	Power and dwell time

AI: ablation index; AF: atrial fibrillation; LSI: lesion size index; PAF: paroxysmal atrial fibrillation; PsAF: persistent atrial fibrillation; RF: radiofrequency; STSF : SmartTouch surround flow.

**Table 3:** Clinical efficiency and efficacy between the HPSD group and CAT group.

Study	Sample size/HPSD population	Power (W)	AF type	Clinical efficiency HPSD group vs. CAT group			Clinical efficacy HPSD group vs. CAT group			Ablation strategy
				Procedure time (min)	RF ablation time (min)	Fluoroscopy time (min)	First-round isolation (%)	Acute PV reconnection (%)	Long term AF recurrence rate (%)	
Bunch et al. [21]	804/402	50	PAF and PsAF	104.3 ± 63.6 vs. 170.8 ± 59.2 (*P* < 0.0001)	—	15.0 ± 8.4 vs. 20.1 ± 18.6 (*P* < 0.0001)	—	—	12.9 vs. 16.2 (*P*=0.19) (1 year) 26.5 vs. 30.7 (*P*=0.23) (3 years)	PVI ± linear ablation
Vassallo et al. [24]	76/41	45–50	PAF and PsAF	106 ± 23vs148 ± 33.6 (*P* < 0.0001)	31.8 ± 11.3 vs. 76.0 ± 33.3 (*P* < 0.0001)	8.8 ± 6.6 vs. 8.5 ± 3.5 (*P*=0.221)	—	—	17.07 vs. 31.42 (*P*=0.14) (1 year)	PVI
Okamatsu et al. [25]	60/20	40–50	PAF and PsAF	—	40 [28–63] vs. 84[272–93] (*P*=0.002)	10 [IQR 8–11] vs. 10 [IQR 8–13] (*P*=0.14)	85 vs. 55 (*P*=0.002)	0 vs. 10 (*P*=0.03)	0 vs. 5 (*P*=0.44) (6 months)	PVI ± CTI ± linear ablation ± CFAE ± SVCI
Berte et al. [26]	174/80	35–45	PAF and PsAF	82 ± 18 vs. 100 ± 22 (*P* < 0.0001)	23±5 vs. 36 ± 11 (*P* < 0.0001)	—	RPV: 83 vs. 84 (*P*=0.79) LPV: 94 vs. 90 (*P*=0.42)	—	18 vs. 17 (*P*=0.93) (6 months)	PVI ± CTI
Kottmaier et al. [27]	197/97	70	PAF	89.5 ± 23.9 vs. 111.15 ± 27.9 (*P* < 0.001)	12.4 ± 3.4 vs. 35.6 ± 12.1 (*P* < 0.0001)	6.3 ± 3.9 vs. 6 ± 3.8 (*P*=0.64)	—	—	18.9 vs. 34.9 (*P* < 0.013) (1 year)	PVI
Pambrun et al. [29]	100/50	40–50	PAF	73.1 ± 18.2 vs. 107.4 ± 21.2 (*P* < 0.001)	13 ± 2.9 vs. 30.3 ± 8.8 (*P* < 0.001)	—	92 vs. 73 (*P* < 0.0001)	2 vs. 17 (*P* < 0.001)	—	PVI
Castrejón-Castrejón et al. [30]	95/48	50–60	PAF and PsAF	106 ± 33 vs. 120 ± 45 (*P*=0.24)	17±3 vs. 34 ± 7 (*P* < 0.001)	7 ± 6 vs. 30 ± 16 (*P* < 0.001)	57 vs. 39 (*P*=0.01)	5 vs. 8 (*P*=0.56)	—	PVI ± CTI ± linear ablation
Ejima et al. [31]	120/60	50	PAF	108.9 ± 22.6 vs. 123.6 ± 27.1 (*P*=0.03)	15.6 ± 5.6 vs. 31.6 ± 9.9 (*P* < 0.0001)	0.3[IQR 0–6] vs. 9.5[IQR 7.0–12.3] (*P* < 0.0001)	—	62 vs. 78 (*P*=0.046)	11.7 vs. 26.7 (*P*=0.0423) (1 year)	PVI ± SVCI
Yazaki et al. [32]	64/32	50	PAF	115 ± 32 vs. 150 ± 57 (*P*=0.24)	10±3 vs. 24 ± 6 (*P* < 0.0001)	12 ± 9 vs. 13 ± 6 (*P*=0.24)	—	—	28.1 vs. 34.4 (*P*=0.99) (10 months)	PVI ± CTI
Yavin et al. [33]	224/112	45–50	PAF and PsAF	—	17.2[median 17.5; range 14.6 to 29.2] vs. 31.1 [median31; range 24.6 to 70.1] (*P* < 0.001)	—	90.2 vs. 83.0 (*P*=0.03)	—	—	PVI ± CTI ± linear ablation
Shin et al. [34]	150/50	50	PAF and PsAF	108.7 ± 23.1 vs. 135.6 ± 29.5 vs. 161.9 ± 37.9 (30 W vs. 40 W vs. 50 W) (*P* < 0.001)	38.2 ± 14.8 vs. 52.3 ± 21.5 vs. 73.1 ± 30.5 (30 W vs. 40 W vs. 50 W) (*P* < 0.001)	9.7 ± 4.1 vs. 11.0 ± 3.0 vs. 12.5 ± 3.6 (30 W vs. 40 W vs. 50 W) (*P*=0.001)		14.0 vs. 10.0 vs. 16.0 (30 W vs. 40 W vs. 50 W) (*P*=0.618)	14.0 vs. 16.0 vs. 18.0 (*P*=0.862) (1 year)	PVI ± CTI ± linear ablation

AF: atrial fibrillation; CFAE: complex fractionated atrial electrograms; CAT: conventional ablation therapy; CTI: cavotricuspid isthmus; HPSD: high-power short-duration; IQR: interquartile range; PAF: paroxysmal atrial fibrillation; PsAF: persistent atrial fibrillation; PV: pulmonary vein; PVI: pulmonary vein isolation; RF: radiofrequency; SVCI: superior vena cava ablation.

## Data Availability

The data used to support the findings of this study are included within the review.
